# Supplementary material for: Sex disparate gut microbiome and metabolome perturbations precede disease progression in a mouse model of Rett syndrome
Source: Commun Biol. 2021 Dec 16;4:1408. doi: 10.1038/s42003-021-02915-3 (PMC8677842; doi:10.1038/s42003-021-02915-3)
Supplement: Supplementary file 2 — Supplementary Information [file 42003_2021_2915_MOESM2_ESM.pdf]

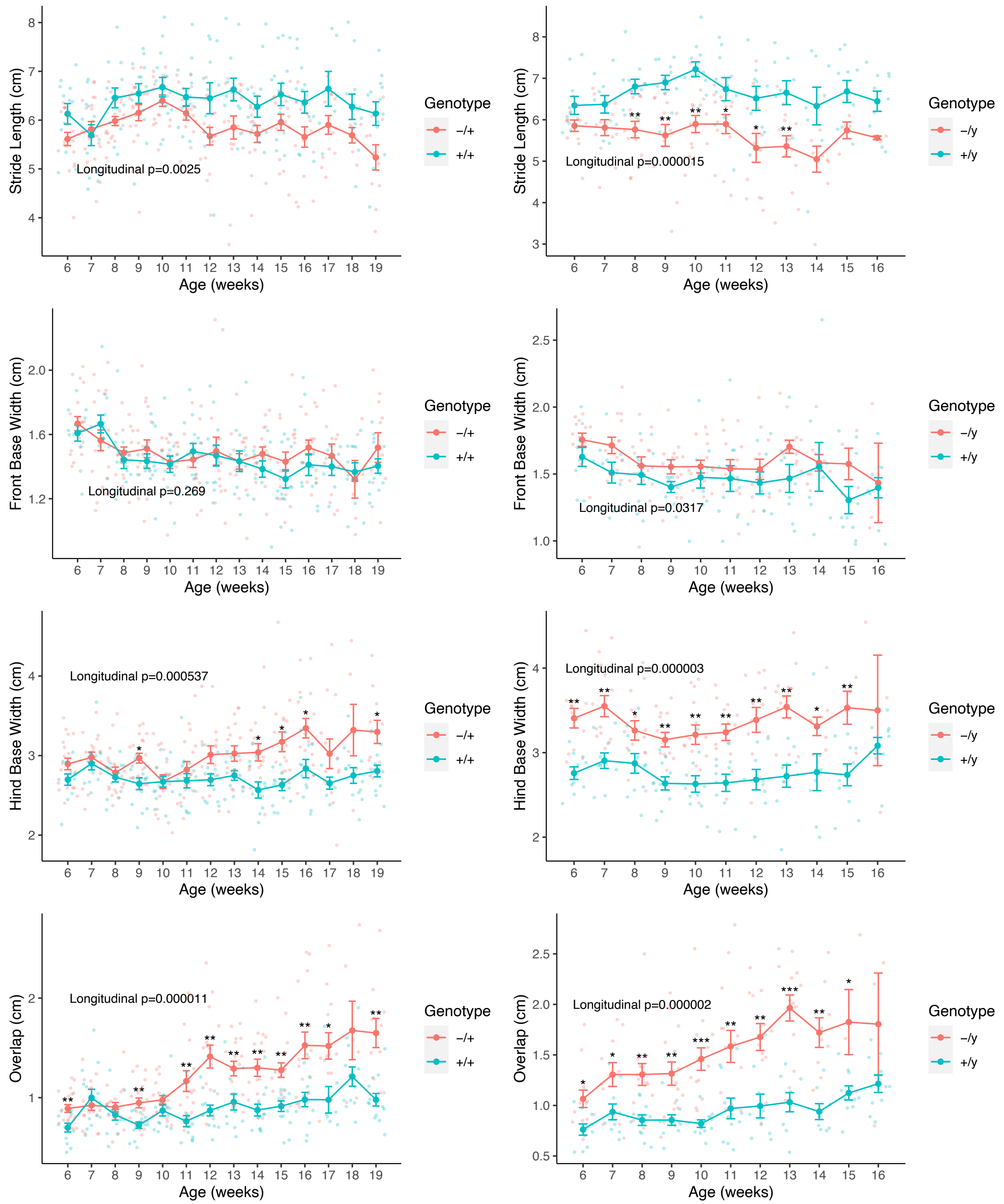

Supplementary Figure 1. Longitudinal gait analysis. Comparison of four different measures of gait across genotypes: 1) stride length, 2) front base width (distance between the two front legs while walking in cm), 3) hind base width (distance between

the two hind legs while walking in cm), and 4) overlap distance (distance between front and hind paws while walking). Dots represent the mean and error bars are +/- standard error of the mean (SEM). N=11-19/genotype/sex. *FDR<0.05, **FDR<0.01, ***FDR<0.001 in mutant vs. control, cross-sectionally. Longitudinal p-values represent the overall association between mutant and wild-type mice across disease course using linear mixed effects models.

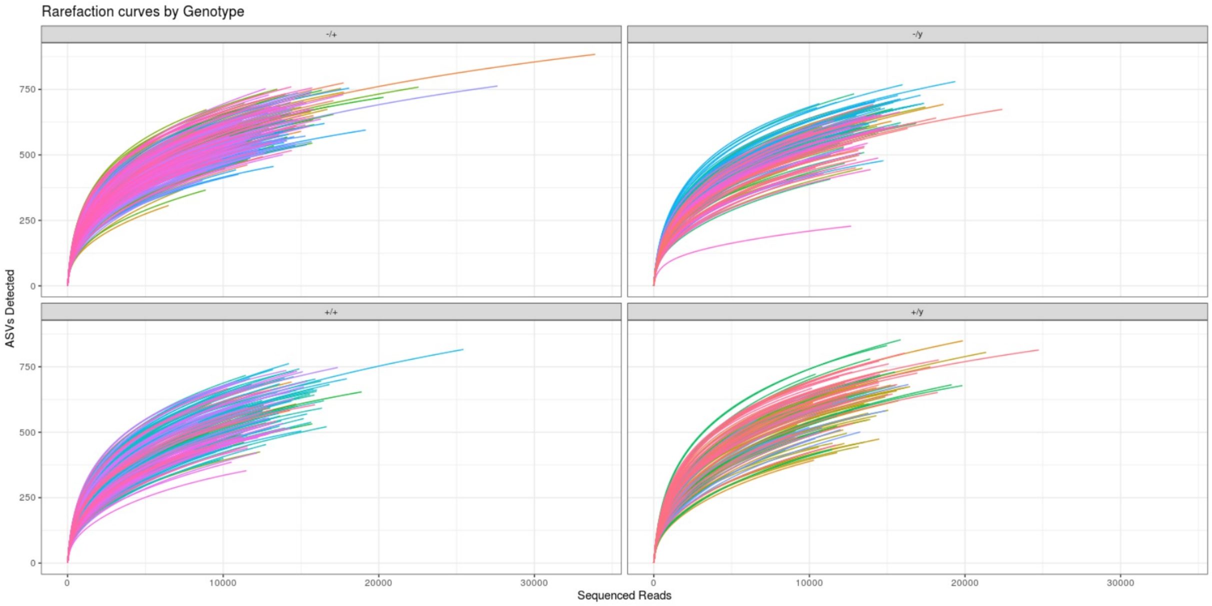

Supplementary Figure 2. Rarefaction curves by sex and genotype. Rarefaction curves show the number of Amplicon Sequence Variants (ASVs) detected as a function of sequencing depth. Each line represents an individual sample. N=11-19/genotype/sex/time point.

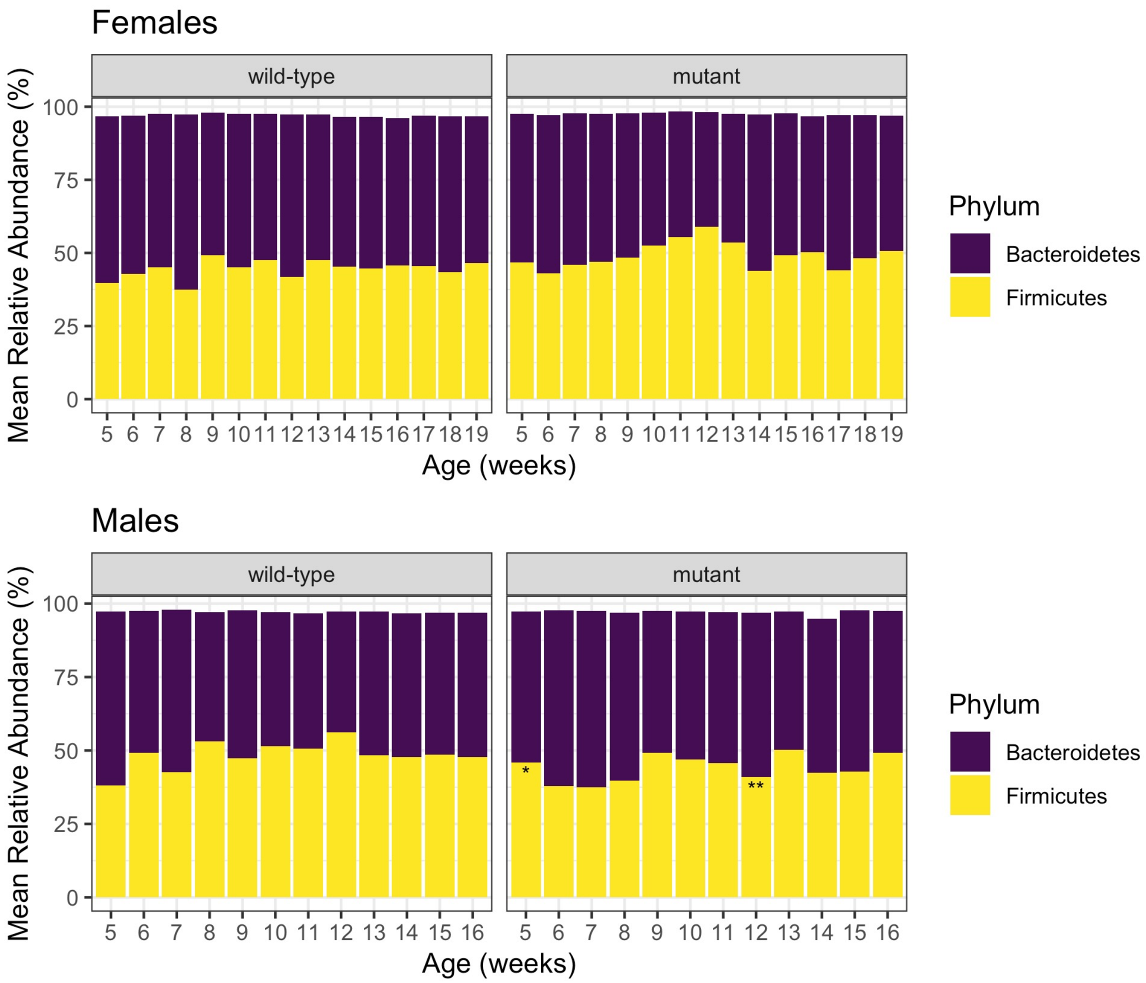

Supplementary Figure 3. Relative abundance of major phyla in fecal samples by sex and genotype. Mean relative abundance for the two major phyla in the mouse gut microbiome (*Firmicutes* and *Bacteroidetes*) represented by stacked bar plots at each collection time point, stratified by sex and genotype. N=11-19/genotype/sex/time point. *FDR<0.05, **FDR<0.01 in mutant vs. wild-type cross-sectionally.

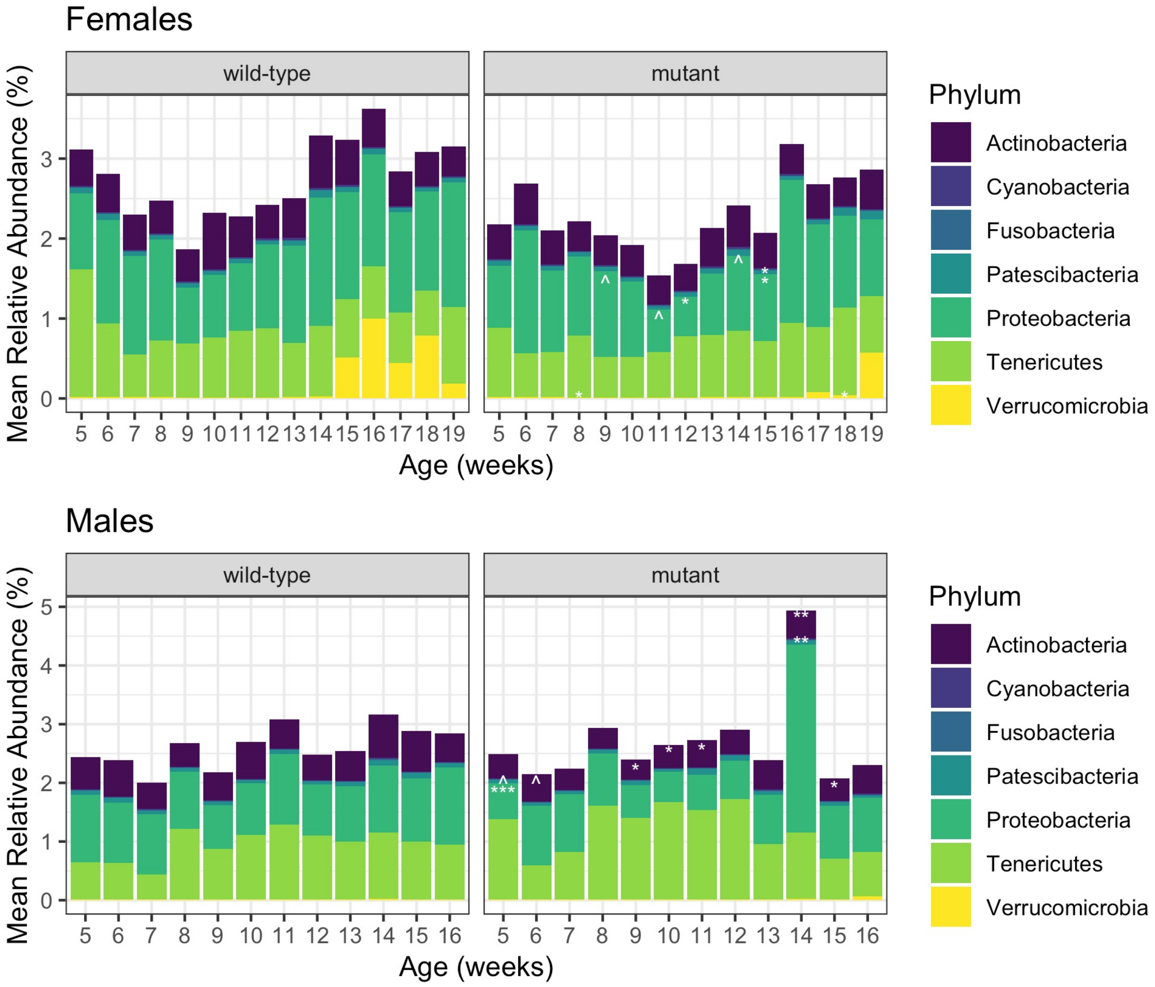

Supplementary Figure 4. Relative abundance of minor phyla in fecal samples by sex and genotype. Mean relative abundance for minor phyla in the mouse gut microbiome (*Actinobacteria*, *Cyanobacteria*, *Fusobacteria, Patescibacteria, Proteobacteria, Tenericutes*, and *Verrucomicrobia*) represented by stacked bar plots at each collection time point, stratified by sex and genotype. N=11-19/genotype/sex/time point. ^FDR<0.10, *FDR<0.05, **FDR<0.01 in mutants vs. wild-type.

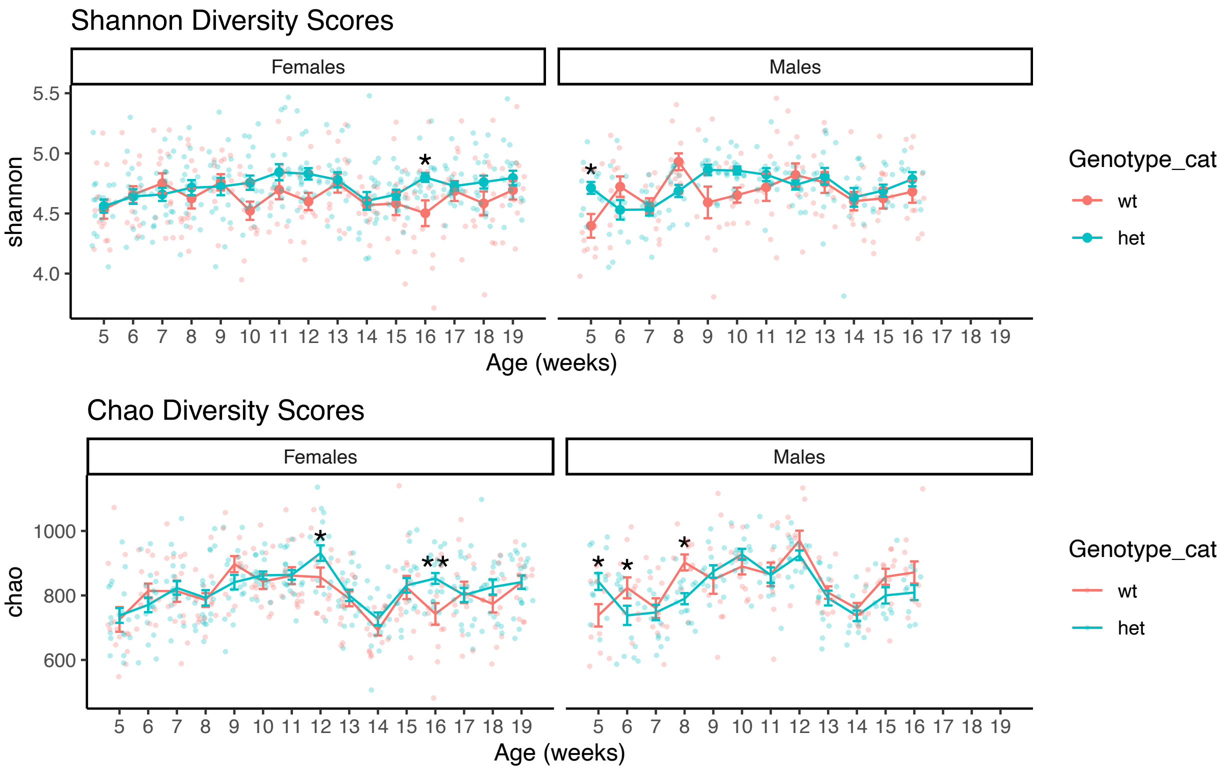

Supplementary Figure 5. Diversity scores across time. Chao and Shannon diversity scores plotted across time for both females (F) and males (M) in mutant vs. wild-type (wt). Dots represent mean score and error bars are +/- standard error of the mean (SEM). N=11-19/genotype/sex/time point. *FDR<0.05, **FDR<0.01 in mutant vs. wt cross-sectionally.

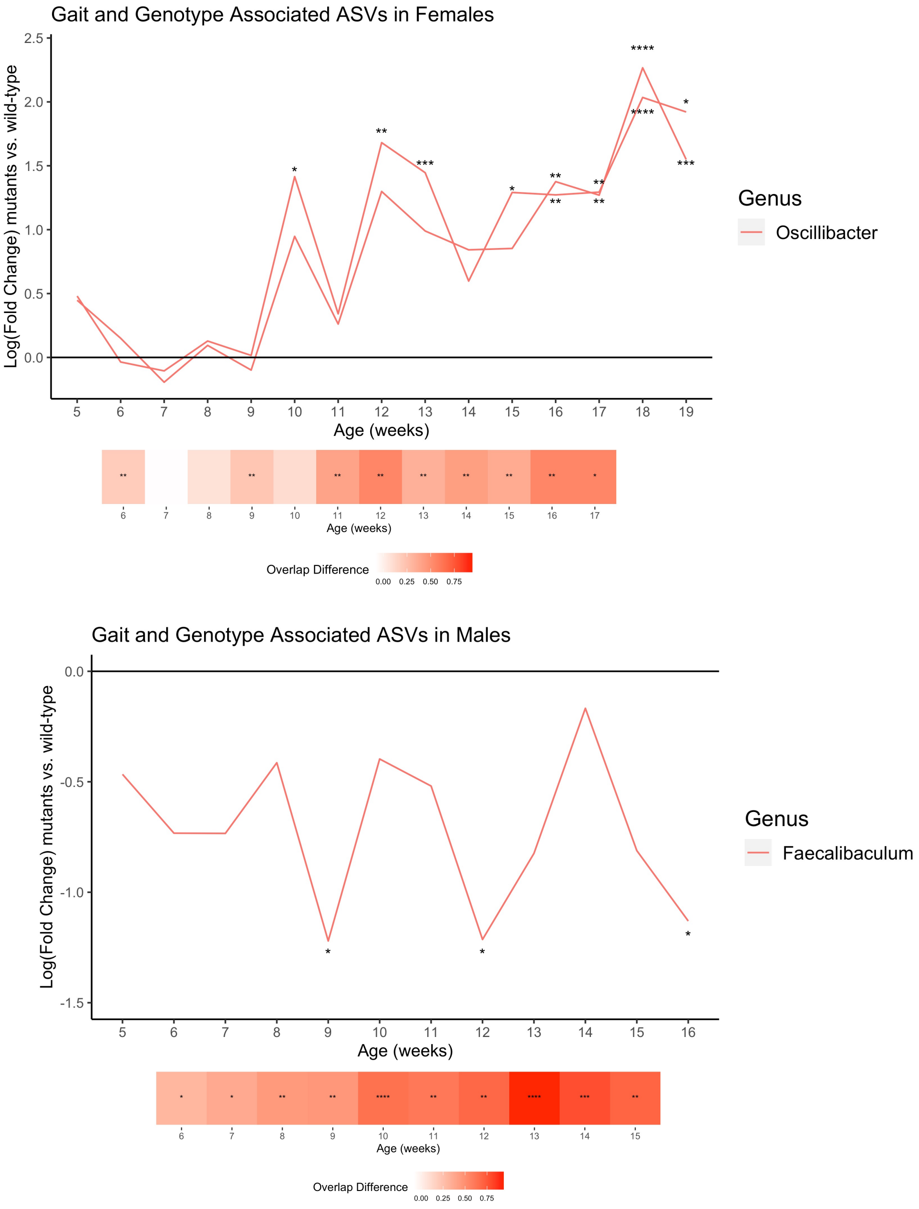

Supplementary Figure 6. Differences in genotype- and gait-associated ASVs in mutant vs. wild-type *Mecp2-e1* mice across disease course. Log(fold change) of mutant vs. wild-type (wt) mice at each time point in ASVs that were significantly associated with gait (overlap distance) and genotype at a minimum of one-quarter of the

time points in both females and males. Each line represents an individual ASV and lines are colored by Genus. Heatmaps at the bottom of each graph are colored red with increasing intensity based on the difference in phenotype measure by genotype at each time point as reported in Figure 1. N=11-19/genotype/sex. *FDR<0.05, **FDR<0.01, ***FDR<0.001 in *Mecp2-e1* mutant vs. wild-type mice cross-sectionally at each time point.

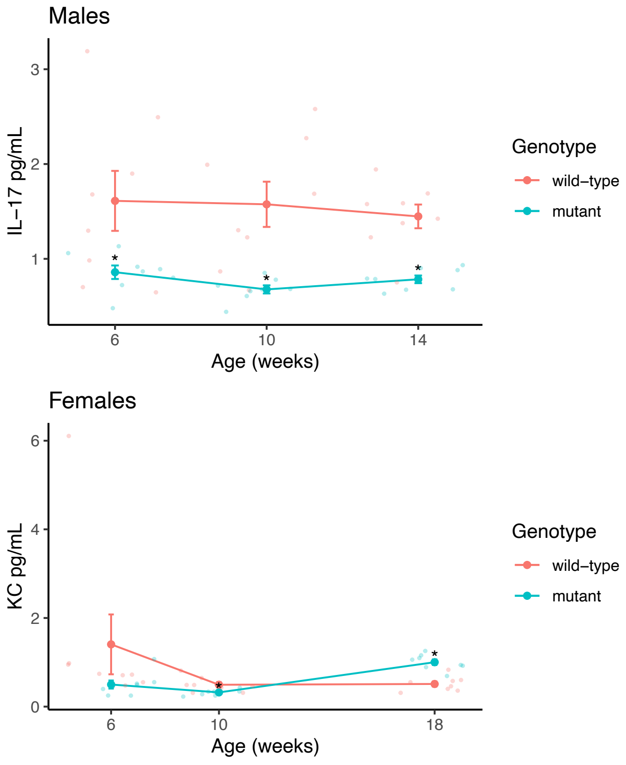

Supplementary Figure 7. Additional fecal cytokine levels across disease course that are significantly different between *Mecp2-e1* mutants vs. wild-type controls. Comparison of fecal interleukin-17 (IL-17) and keratinocytes-derived chemokine (KC), levels between *Mecp2-e1* mutant and wild-type (wt) mice longitudinally and cross-

sectionally across disease course (at 6, 10, and 18 weeks in females and 6, 10, and 14 weeks in males). Analyses were stratified by sex and controlled for within-litter effects. Dots represent the mean and error bars are +/- standard error of the mean (SEM). ^FDR<0.10, *FDR<0.05 in mutant vs. wild-type controls cross-sectionally. N = 6-8/genotype/sex. Detailed Ns for each time point are included in **Supplementary Table 12**.

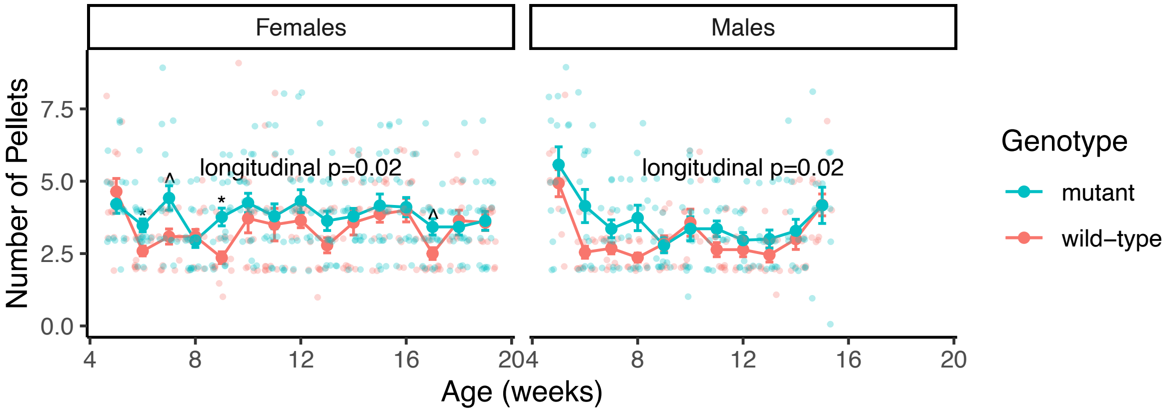

Supplementary Figure 8. Differences in fecal pellet passage rate in mutant vs. wild-type *Mecp2-e1* mice across disease course. Fecal pellet passage rate was measured as the number of fecal pellets passed in a five minute period. Dots represent the mean and error bars are +/- standard error of the mean (SEM). N=11-19/genotype/sex. *FDR<0.05, **FDR<0.01, ***FDR<0.001 in mutant vs. control, cross-sectionally. Longitudinal p-values represent the overall association between mutant and wild-type mice across disease course using linear mixed effects models.

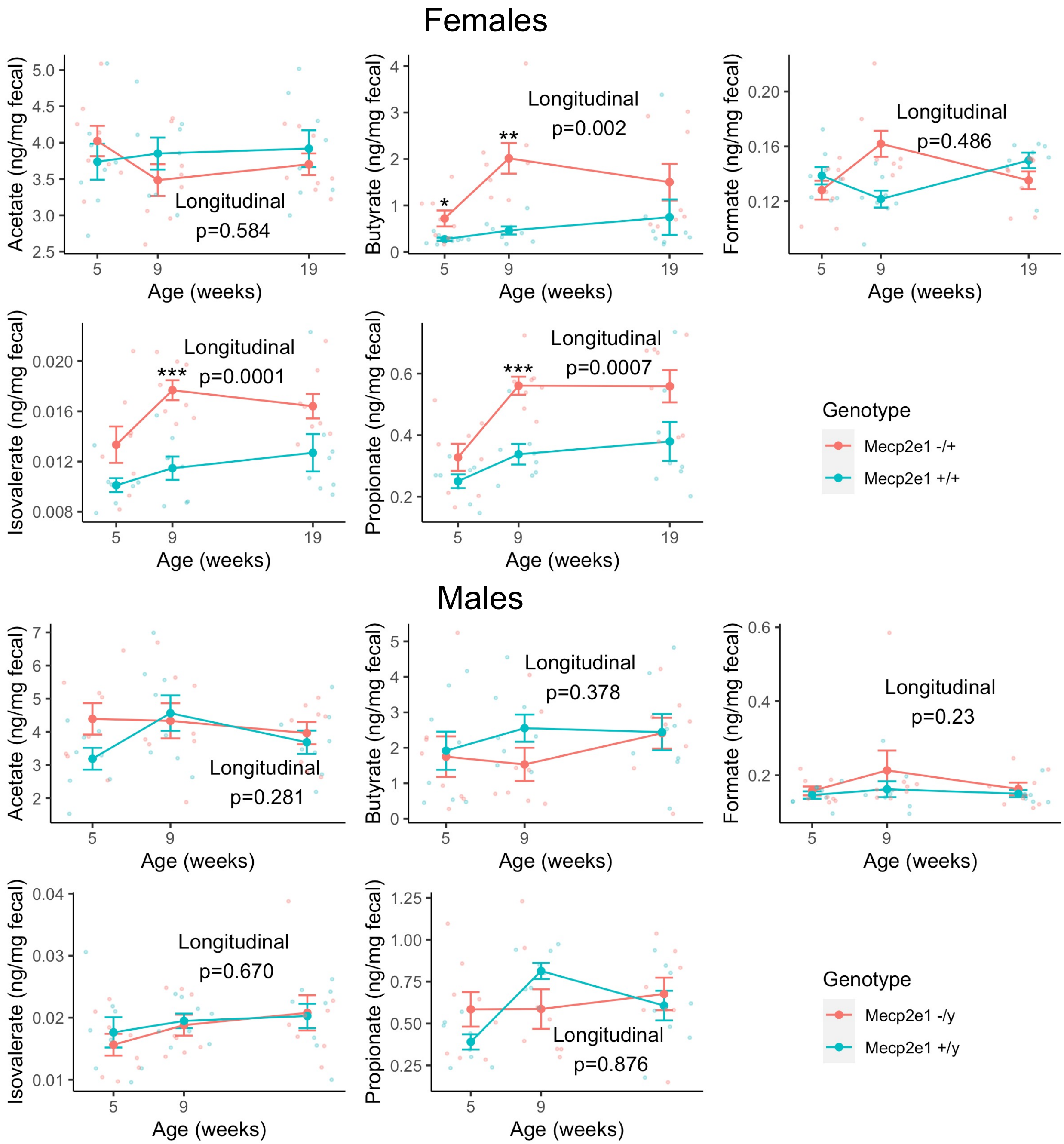

Supplementary Figure 9. Fecal SCFA levels across age in *Mecp2-e1* mutant vs. wild-type females and males. Each dot represents the mean of each group at each time point and error bars are +/- standard error of the mean (SEM). N=8/genotype/sex. *FDR<0.05, **FDR<0.01 in *Mecp2-e1* mutant vs. wild-type cross-sectionally.

Longitudinal p-values represent the overall association between mutant and wild-type mice across disease course using linear mixed effects models controlling for within-litter effects and repeated measures.

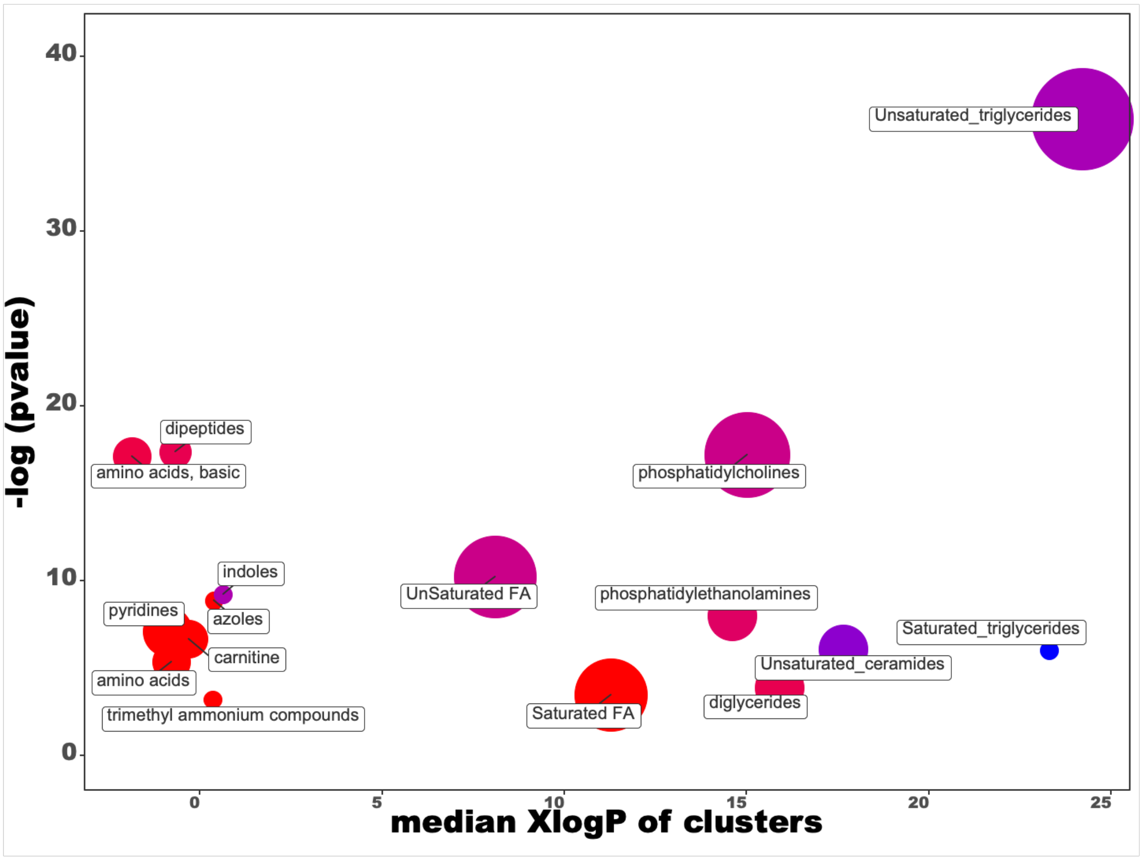

Supplementary Figure 10. Fecal metabolite clusters enriched in female Mecp2-e1 -/+ vs. +/+ mice. The y-axis is -log(p-value) resulting in the most statistically significant clusters being placed higher on the graph. Median XlogP (x-axis) of clusters is the cluster order on the cluster similarity tree generated by ChemRICH. Cluster colors give the proportion of increased or decreased compounds (red = increased, blue = decreased), and the size of each circle is determined by the total number of metabolites in each cluster.

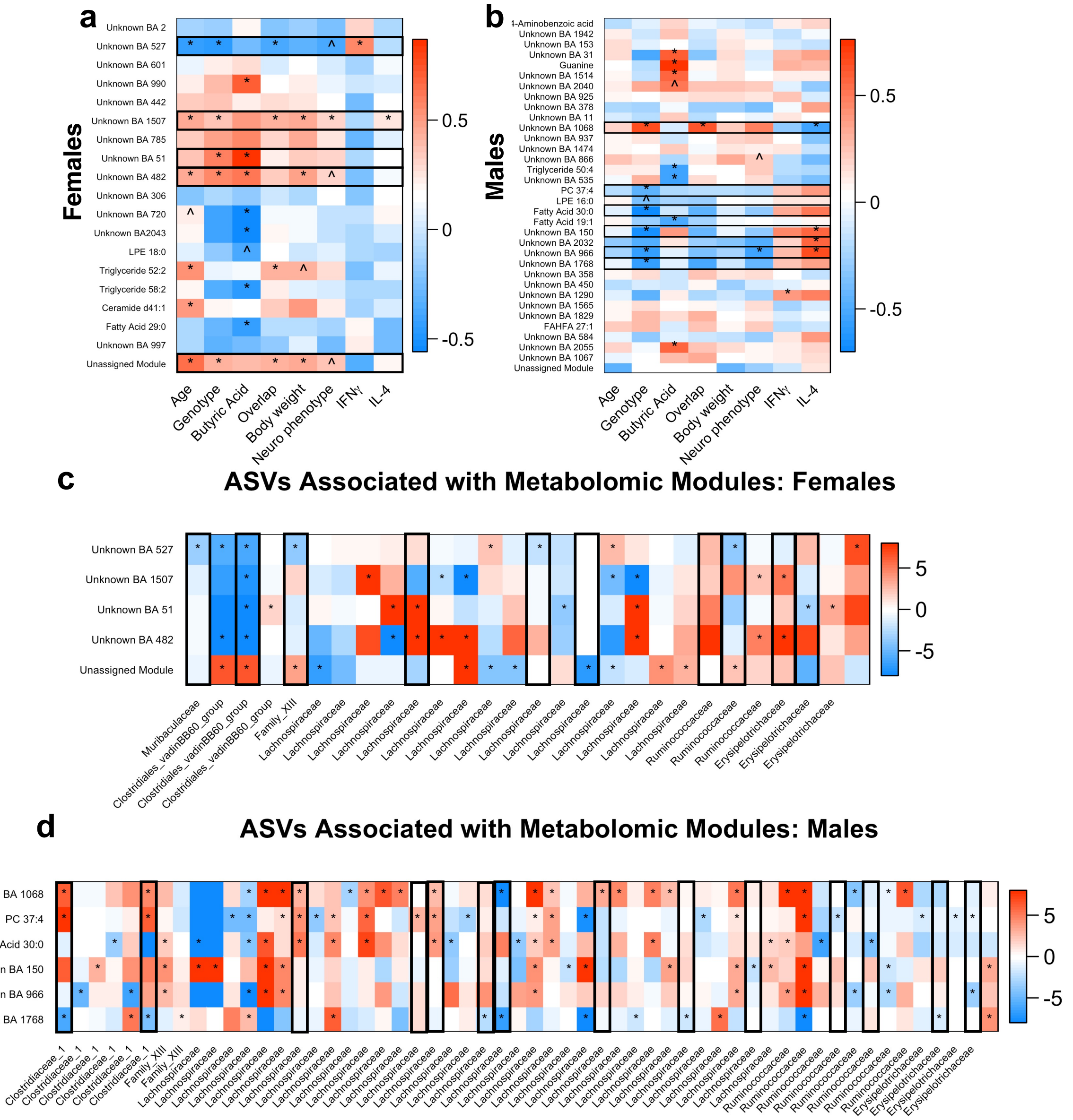

Supplementary Figure 11. Relationships between the fecal metabolome and genotype, phenotype, and the fecal microbiome. Heatmaps depicting relationships between fecal metabolomic modules as constructed through weighted gene co-network analysis (WGCNA) and age, genotype, and phenotypes in females (**a**) and males (**b**). Modules were designated based on a “hub” metabolite as identified using WGCNA.

Blocks are colored by correlation coefficients with red representing positive correlations and blue representing negative correlations. Black boxes denote modules that were significantly associated with genotype (FDR<0.05). Statistical analyses evaluating relationships between modules and traits were carried out via linear mixed effects models to control for within-litter effects and repeated measures. Relationships between genotype-associated metabolomic modules and fecal microbiota in females (**c**) and males (**d**) are depicted via heatmaps with blocks colored by log(fold change). Black boxes denote microbiota that were also associated with at least one phenotype. Statistical analyses were carried out using limma controlling for within-litter effects and repeated measures. The only ASVs shown were those that were statistically significantly associated with at least one genotype-associated metabolomics module. N=8/genotype/sex. *FDR<0.05, ^FDR<0.10.

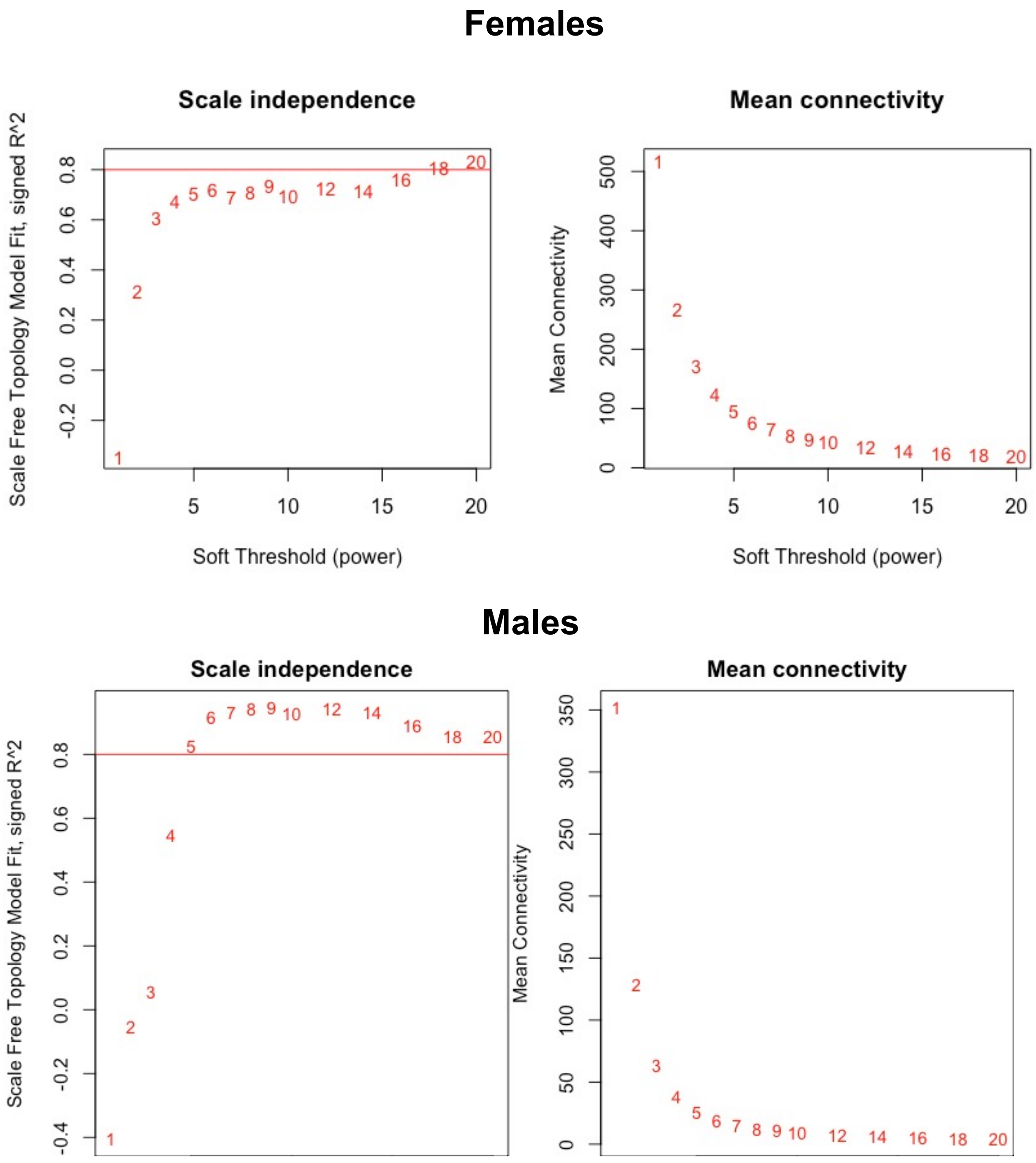

Supplementary Figure 12. Fecal metabolite scale independence and mean connectivity plots for WGCNA analysis in females and males. The soft threshold plotted against scale free topology model fit (R^2) is used to determine a soft power threshold for network construction. The smallest value at which the scale free topology

model R2 is at least 0.8 is selected as the soft power threshold. Mean connectivity plots examine the network connectivity plotted against different soft power thresholds.

Females

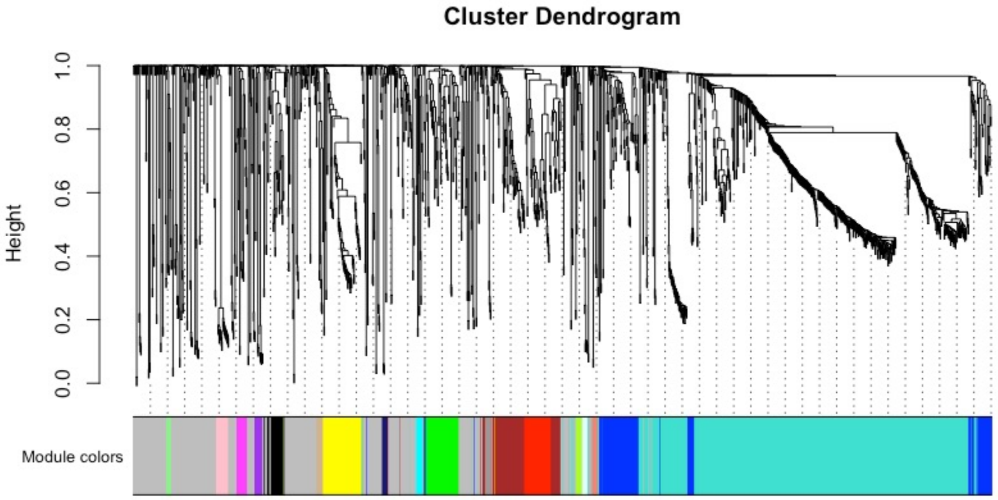

Males

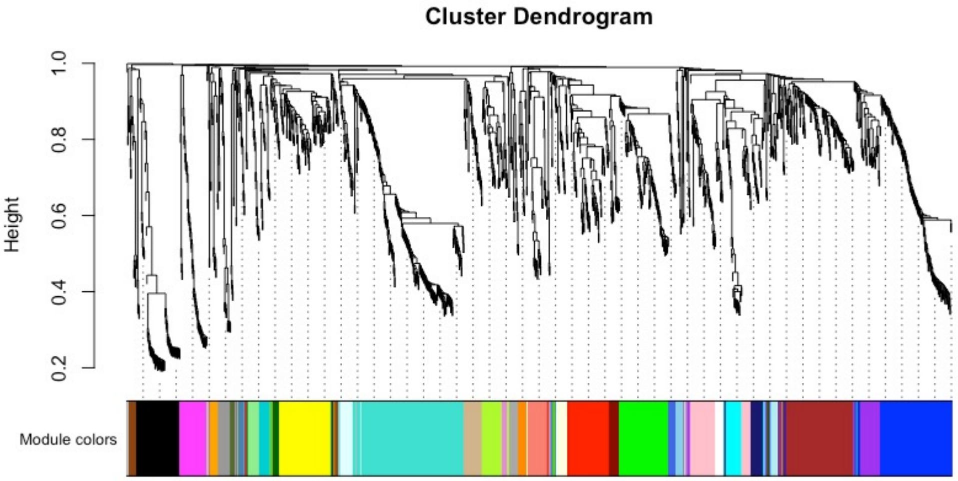

Supplementary Figure 13. Fecal metabolite modules represented as cluster dendrograms. Cluster dendrograms for WGCNA modules depict the clustering of metabolites and how they fit within each module.
