# Supplementary material for: Sex disparate gut microbiome and metabolome perturbations precede disease progression in a mouse model of Rett syndrome
Source: Commun Biol. 2021 Dec 16;4:1408. doi: 10.1038/s42003-021-02915-3 (PMC8677842; doi:10.1038/s42003-021-02915-3)
Supplement: Supplementary file 3 — Description of Additional Supplementary Files [file 42003_2021_2915_MOESM3_ESM.pdf]

Description of Additional Supplementary Files

File name: Supplementary Data

Description:

Supplementary Data 1: Longitudinal analysis of relative abundance of microbiota classes.

Supplementary Data 2: Main Effect of Genotype ASVs in Females.

Supplementary Data 3: Main Effect of Genotype ASVs in Males.

Supplementary Data 4: Genotype x Age Interaction Effect on ASVs in Females.

Supplementary Data 5: Genotype x Age Interaction Effect on ASVs in Males.

Supplementary Data 6: ASVs associated with body weight in females.

Supplementary Data 7: ASVs associated with gait (overlap distance) in females.

Supplementary Data 8: ASVs associated with neurophenotyping score (females).

Supplementary Data 9: ASVs associated with body weight in males.

Supplementary Data 10: ASVs associated with gait (overlap distance) in males.

Supplementary Data 11: ASVs associated with neurophenotyping score in males.

Supplementary Data 12: Analysis of fecal cytokines in *Mecp2-e1* mutant vs. wild-type mice.

Supplementary Data 13: Fecal metabolite clustering by ChemRICH.

Supplemental Data 14: Metabolite module membership in females.

Supplemental Data 15: Metabolite module membership in males.

Supplementary Data 16: Comparison of brain lipids in female *Mecp2-e1* mutants vs. wild-type.

Supplementary Data 17: Brain lipid clusters significantly altered (FDR<0.10) in *Mecp2-e1* -/+ vs. *Mecp2-e1* +/+ females.

Supplementary Data 18: Brain lipid clustering via ChemRICH.
