# Supplementary material for: Sex disparate gut microbiome and metabolome perturbations precede disease progression in a mouse model of Rett syndrome
Source: Commun Biol. 2021 Dec 16;4:1408. doi: 10.1038/s42003-021-02915-3 (PMC8677842; doi:10.1038/s42003-021-02915-3)
Supplement: Supplementary file 5 — Reporting Summary [file 42003_2021_2915_MOESM5_ESM.pdf]

nature research

Corresponding author(s): Janine LaSalle

Last updated by author(s): October 29, 2021

Reporting Summary

Nature Research wishes to improve the reproducibility of the work that we publish. This form provides structure for consistency and transparency in reporting. For further information on Nature Research policies, see our Editorial Policies and the Editorial Policy Checklist.

Statistics

For all statistical analyses, confirm that the following items are present in the figure legend, table legend, main text, or Methods section.

n/a	Confirmed	
☐	☒	The exact sample size (*n*) for each experimental group/condition, given as a discrete number and unit of measurement
☐	☒	A statement on whether measurements were taken from distinct samples or whether the same sample was measured repeatedly
☐	☒	The statistical test(s) used AND whether they are one- or two-sided<br>*Only common tests should be described solely by name; describe more complex techniques in the Methods section.*
☐	☒	A description of all covariates tested
☐	☒	A description of any assumptions or corrections, such as tests of normality and adjustment for multiple comparisons
☐	☒	A full description of the statistical parameters including central tendency (e.g. means) or other basic estimates (e.g. regression coefficient) AND variation (e.g. standard deviation) or associated estimates of uncertainty (e.g. confidence intervals)
☐	☒	For null hypothesis testing, the test statistic (e.g. *F*, *t*, *r*) with confidence intervals, effect sizes, degrees of freedom and *P* value noted<br>*Give P values as exact values whenever suitable.*
☒	☐	For Bayesian analysis, information on the choice of priors and Markov chain Monte Carlo settings
☐	☒	For hierarchical and complex designs, identification of the appropriate level for tests and full reporting of outcomes
☐	☒	Estimates of effect sizes (e.g. Cohen's *d*, Pearson's *r*), indicating how they were calculated

Our web collection on statistics for biologists contains articles on many of the points above.

Software and code

Policy information about availability of computer code

Data collection	dbcAmplicons (https://github.com/msettles/dbcAmplicons version 0.8.5)
Data analysis	Metaboanalyst version 4.0<br>R version 3.6.3<br>Bioconductor version 3.10<br>HTStream (https://github.com/s4hts/HTStream version 1.2.0)<br>DADA2 version 1.14.1<br>WGCNA version 1.69<br>Limma version 3.42.2

For manuscripts utilizing custom algorithms or software that are central to the research but not yet described in published literature, software must be made available to editors and reviewers. We strongly encourage code deposition in a community repository (e.g. GitHub). See the Nature Research guidelines for submitting code & software for further information.

Data

Policy information about availability of data

All manuscripts must include a data availability statement. This statement should provide the following information, where applicable:

- Accession codes, unique identifiers, or web links for publicly available datasets
- A list of figures that have associated raw data
- A description of any restrictions on data availability

Data are available within Supplementary Data Files, Supplementary Tables, and on Figshare at https://doi.org/10.6084/m9.figshare.16906597.v1. Additional data will be made available upon reasonable request. Code used in the bioinformatic analysis of microbiome data is available at https://github.com/msettles/

dbcAmplicons version 0.8.5.

Field-specific reporting

Please select the one below that is the best fit for your research. If you are not sure, read the appropriate sections before making your selection.

[x] Life sciences [ ] Behavioural & social sciences [ ] Ecological, evolutionary & environmental sciences

For a reference copy of the document with all sections, see nature.com/documents/nr-reporting-summary-flat.pdf

Life sciences study design

All studies must disclose on these points even when the disclosure is negative.

Sample size	58 total mice representing 2 sexes and 4 genotypes for groups of 11-19 mice were studied from weaning up until 22 weeks of age. Power analysis was used to establish that a sample size of 12 was sufficient to observe significant weight, motor and disease phenotypes.
Data exclusions	For microbiome analysis 6 out of 787 total 16S amplicon sequencing read samples were excluded due to reduced ASV diversity below 250 and were discarded by a pre-established exclusion criteria.
Replication	This study represents a replication of the weight, gait and disease phenotypes of Mecp2e1 deficient mice.
Randomization	Samples were organized into groups based on genotype, sex and age of the mice that the samples were derived from.
Blinding	For the weight, gait and disease phenotyping the data was collected in a blinded manner.

Reporting for specific materials, systems and methods

We require information from authors about some types of materials, experimental systems and methods used in many studies. Here, indicate whether each material, system or method listed is relevant to your study. If you are not sure if a list item applies to your research, read the appropriate section before selecting a response.

Materials & experimental systems

n/a	Involved in the study	
[x]	[ ]	Antibodies
[x]	[ ]	Eukaryotic cell lines
[x]	[ ]	Palaeontology and archaeology
[ ]	[x]	Animals and other organisms
[x]	[ ]	Human research participants
[x]	[ ]	Clinical data
[x]	[ ]	Dual use research of concern

Methods

n/a	Involved in the study	
[x]	[ ]	ChIP-seq
[x]	[ ]	Flow cytometry
[x]	[ ]	MRI-based neuroimaging

Animals and other organisms

Policy information about studies involving animals; ARRIVE guidelines recommended for reporting animal research

Laboratory animals	Mus musculus, C57BL6/J strain of both sexes were studied from weaning to up to 22 weeks.
Wild animals	*Provide details on animals observed in or captured in the field; report species, sex and age where possible. Describe how animals were caught and transported and what happened to captive animals after the study (if killed, explain why and describe method; if released, say where and when) OR state that the study did not involve wild animals.*
Field-collected samples	*For laboratory work with field-collected samples, describe all relevant parameters such as housing, maintenance, temperature, photoperiod and end-of-experiment protocol OR state that the study did not involve samples collected from the field.*
Ethics oversight	All studies conform to NIH policies and ethical approval was obtained from the University of California Davis IACUC under protocol #20621

Note that full information on the approval of the study protocol must also be provided in the manuscript.
